# Comparing the Environmental Influences and Community Assembly of Protist Communities in Two Anthropogenic Coastal Areas

**DOI:** 10.3390/microorganisms12081618

**Published:** 2024-08-08

**Authors:** Wenwen Qiao, Hongbo Li, Jinyong Zhang, Xiaohan Liu, Ruofei Jin, Hongjun Li

**Affiliations:** 1 Key Laboratory of Industrial Ecology and Environmental Engineering (Ministry of Education), School of Environmental Science and Technology, Dalian University of Technology, Dalian 116024, China; wenwen020416@163.com; 2 State Environmental Protection Key Laboratory of Coastal Ecosystem, National Marine Environmental Monitoring Center, Dalian 116023, China; hbli@nmemc.org.cn (H.L.); jyzhang@nmemc.org.cn (J.Z.); xhliu@nmemc.org.cn (X.L.)

**Keywords:** anthropogenic stresses, protist, estuarine ecosystems, community assembly, functional traits

## Abstract

Anthropogenic stresses are intensively affecting the structure and function of microbial communities in coastal ecosystems. Despite being essential components of coastal ecosystems, the environmental influences and assembly processes of protist communities remain largely unknown in areas with severe disturbance. Here, we used 18S rRNA gene high-throughput sequencing to compare the composition, assembly process, and functional structure of the protist communities from the coastal areas of the Northern Yellow Sea (NYS) and the Eastern Bohai Sea (EBS). These two areas are separated by the Liaodong Peninsula and experience different anthropogenic stresses due to varying degrees of urbanization. We detected significant differences between the protist communities of the two areas. Environmental and geographic factors both influenced the composition of protist communities, with environmental factors playing a greater role. The neutral community model indicated that the assembly of protist communities was governed by deterministic processes, with stochastic processes having a stronger influence in the EBS area compared to the NYS area. The phototrophic and consumer communities, influenced by different environmental factors, differed significantly between the two areas. Our results provide insights into the biogeography and assembly of protist communities in estuaries under anthropogenic stresses, which may inform future coastal management.

## 1. Introduction

Estuaries are critical interfaces between terrestrial and marine ecosystems, harboring complex biological communities [1]. Heterogeneity in physical and chemical conditions between seawater and freshwater creates natural gradients in estuarine environments [2], resulting in highly diverse microbial communities that exhibit significant heterogeneity in diversity, abundance, and composition [3,4]. However, incremental urbanization and industrialization during past decades has negatively affected coastal ecosystems [5], and many estuaries face issues such as eutrophication, hypoxia, and excessive heavy metal loads [6,7]. Predicting the microbial community dynamics and the impact of human activities is urgently needed to provide theoretical support for subsequent biodiversity conservation and estuarine environmental management [8].

Microbial community assembly processes in aquatic systems and the effects of various factors on such processes are central topics in microbial biogeography [9,10]. The metacommunity concept states that deterministic and stochastic processes are primarily responsible for the spatial turnover of natural communities [11]. Neutral theory identifies stochastic processes such as microbial births, immigration from a regional pool of species, and speciation as drivers of microbial community assembly [12,13]. Based on ecological niche theory, environment-related deterministic processes such as homogeneous, heterogeneous selection and biotic interactions play a crucial role in assembly processes [14]. Furthermore, increased environmental stresses caused by human activities have been shown to promote the contribution of deterministic processes in microbial community assembly, and especially elevated nutrients can cause strong environmental selection and thus affect community assembly.

Protists are a highly diverse group of eukaryotic microbes responsible for numerous important ecological and biogeochemical activities in terrestrial and aquatic environments [15]. They play multiple ecological roles in food webs, acting as decomposers, producers, and consumers. Traditional morphological identification of protists is both laborious and time-consuming and requires a high level of expertise. Alternatives to the traditional techniques are crucial to meet the need for large-scale biodiversity monitoring. Environmental DNA (eDNA) metabarcoding approaches based on high-throughput sequencing have been proposed for biomonitoring. Despite being the largest portion of eukaryotic richness, molecular investigations have revealed that most of the taxonomic diversity of aquatic protists remains undescribed [16]. Previous studies have shown that protists reproduce rapidly and are sensitive to human activities and environmental changes, with these responses probably being more pronounced than those of bacteria and fungi [17,18,19]. Although some researchers have explored the mechanisms of protist community assembly processes in aquatic environments, most have studied individual water environments, and few comparative studies of protist communities in different areas have been conducted.

To address this knowledge gap, we compared the protist community profiles in two coastal areas subjected to different levels of anthropogenic stresses. We aimed to determine: (1) the differences in diversity, abundance, and composition of protist communities in the estuaries of the two coastal areas; (2) the processes that influence the assembly of protist communities; and (3) the influence of environmental variables on different functional groups of protists. The results of our study improve our knowledge of microbe-driven ecosystem processes and functions in coastal areas under anthropogenic disturbance.

## 2. Materials and Methods

### 2.1. Study Areas and Sampling Collection

**Figure 1:**
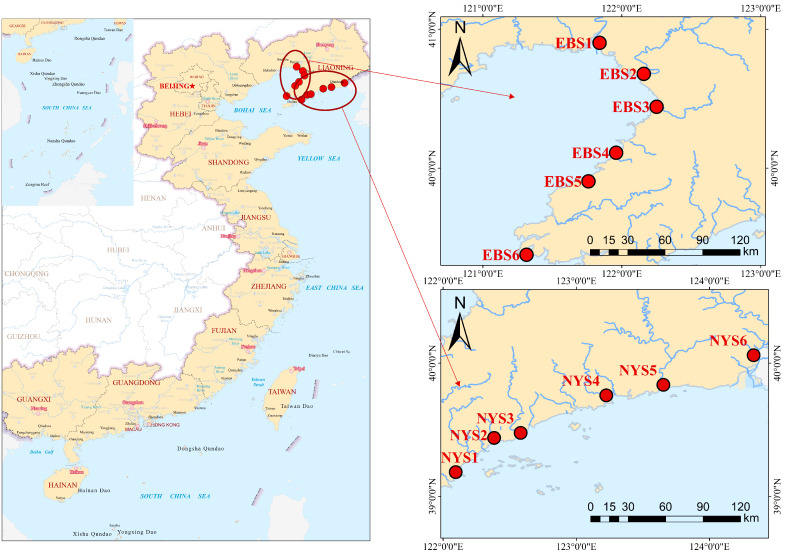
The positions of the sampling stations in the estuaries of the EBS and the NYS.

We selected two contrasting habitats under different anthropogenic stress levels to explore the responses of protist communities to anthropogenic perturbations. The East Bohai Sea (EBS) is located in a typical semi-enclosed shallow sea. The EBS has poor water exchange conditions, which results in longer water residence time and retention duration for pollutants [20]. The EBS coast is industrialized and densely populated, leading to a large amount of land-based source pollutants being discharged into the sea. The North Yellow Sea (NYS) is located on the northeast coast of China, which is directly exposed to the open ocean. The NYS and the EBS are both characterized by temperate monsoon climates, and along with their location in latitude, these minimize the differences in the effects of temperature on protist communities. Water samples were collected in August 2021, located between the geographical coordinates of 121°18′–124°19′ E and 39°10′–40°54′ N. Taking into account the geographic features of the Yellow Sea and the Bohai Sea, we selected 12 estuaries for sampling, with 6 chosen from each area. Within each estuary, we collected samples from 1 sampling station, leading to a total of 12 sampling stations. Water samples were collected in triplicates at each sampling station to ensure the robustness and reproducibility of the data (Figure 1). We collected 2 L of water from a depth of 0.5 m three times at each station. A total of 36 water samples were collected. The samples were stored in sterile plastic containers at 4 °C. Each sample was processed within 24 h of collection. The processing entailed vacuum filtration through sterile membranes with a pore size of 0.22 μm. The filters were immediately frozen at −20 °C and transported to the laboratory, where they were stored at −80 °C until further analysis [21]. We extracted DNA from the samples within 24 h of collection to ensure the preservation of the genetic material.

### 2.2. Measurement of Environmental Variables

Several environmental variables were measured to assess the local conditions. The temperature, salinity, dissolved oxygen (DO), and pH of the water were measured using YSI1001 (YSI Inc., Yellow Springs, OH, USA), and directly in the sampling sites. To further analyze the chemical composition of the water samples, 1 L of each collected sample was brought back to the laboratory. Within 24 h, standard methods were used to determine the concentrations of key nutrients [22]. The concentrations of ammonium ion (NH_4_^+^), nitrite (NO_2_^−^), nitrate (NO_3_^−^), and phosphate (PO_4_^3–^) were quantified to understand the nutrient dynamics in the sampled estuaries.

### 2.3. DNA Extraction, PCR Amplification, and Sequencing

Total DNA was extracted from samples in the 1.2–20 μm size range for 18S rRNA sequencing using the PowerWater DNA Isolation Kit (Qiagen, Redwood City, CA, USA) following the manufacturer’s instructions [22]. We measured DNA concentration using a NanoDrop spectrophotometer (Thermo Fisher Scientific, Carlsbad, CA, USA), and integrity was verified by 2% agarose gel electrophoresis. The V9 region of the 18S rRNA gene was amplified using primers 1380F (TCCCTGCCHTTTG TACACAC) and 1510R (CCTTCYGCAGGTTCACCTAC) [23]. PCR products were checked by 1.5% agarose gel electrophoresis, then purified, quantified, and pooled for library construction. We assessed library quality using the Agilent Bioanalyzer 2100 system (Santa Clara, CA, USA) and Qubit^®^ 2.0 Fluorometer (Thermo Fisher Scientific, Carlsbad, CA, USA). Sequencing was performed on the Illumina NovaSeq platform (San Diego, CA, USA).

### 2.4. Bioinformatics Processing

Reads with an average Phred score below 20, ambiguous bases, homopolymer runs over six bases, primer mismatches, and sequences shorter than 150 base pairs (bp) were removed [24]. High-quality reads were assigned to samples based on unique barcodes. Reads with overlaps over 10 bp and no mismatches were assembled into tags using FLASH [25]. Tags were assigned to amplicon sequence variants (ASVs) using the DADA2 method in QIIME2 and classified against the PR2 v4.12.0 database [26,27]. Non-target ASVs (fungi, metazoans, angiosperms) were excluded, individual ASVs were culled, and a table of ASV abundance was created.

### 2.5. Statistical Analysis

All data were analyzed, numerically processed, and visualized with R (version 4.0.2). The alpha diversity of protist communities was assessed using the Chao1, Shannon, Pielou_J, and Pd_faith indices in the vegan package (version 2.6-4), with Student’s *t*-test comparing differences between NYS and EBS estuary samples. To determine the effects of geographic region on protist communities, the Bray–Curtis distance was calculated using vegan package, followed by principal coordinates analysis (PCoA) using the ape package (version 5.7), and the adonis test to evaluate regional impacts on protist communities. Differences in regional species pools were analyzed using shared microbial ASVs from all coastal samples. We used Venn analysis in VennDiagram (version 1.7.3) to identify shared and unique ASVs [28]. Key taxa distinguishing NYS and EBS samples were identified through random forest analysis using the randomForest package (version 4.7-1.1). We explored the relationship between protist community composition and environmental and geographic factors using Mantel tests and Procrustes analysis in vegan, with distance-based redundancy analysis (db-RDA) in vegan identifying significant associations. The PCNM variables of db-RDA are spatial variables transformed from latitude and longitude, which can simulate underlying spatial structure [29]. Variation partitioning analysis (VPA) in vegan was used to assess the relative importance of environmental and spatial factors in shaping bacterial and protist communities in the NYS and the EBS. The contribution of stochastic processes to protist community assembly was compared using a neutral community model (NCM) in vegan. We classified protists into consumers, parasites, and phototrophs based on literature databases [30], and used PCoA comparing geographic differences. We also applied Spearman correlation analysis to evaluate relationships between protists and environmental variables.

## 3. Results

### 3.1. Composition and Diversity of Protist Communities

**Figure 2:**
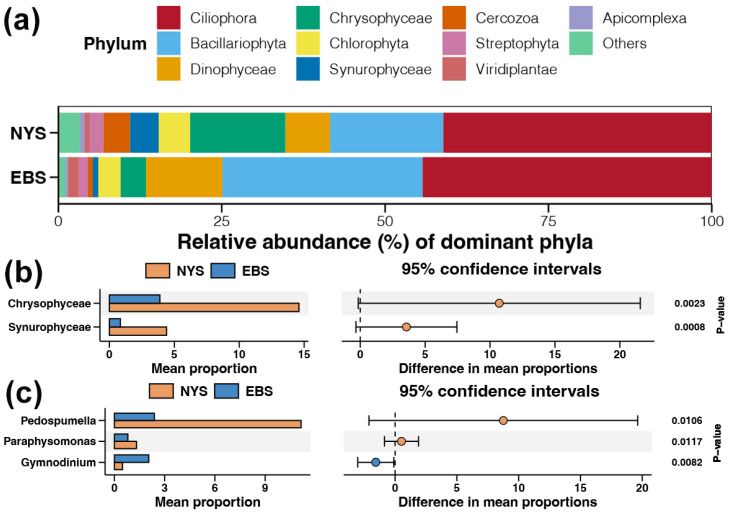
Characteristics of dominant protist taxa in the NYS and EBS. (**a**) Relative abundance of dominant protist phyla in the NYS and EBS estuaries. Protist phyla (**b**) and genera (**c**) showing significant differences between relative abundance in the NYS and EBS.

The most dominant protist phylum detected in samples from both areas was Ciliophora, followed by Bacillariophyta, Dinophyceae, Chrysophyceae, and Chlorophyta (Figure 2a). Among the detected protist phyla, the abundance of Chrysophyceae and Synurophyceae was significantly higher in the NYS estuaries compared to the EBS estuaries (Figure 2b, *p* < 0.01). The most dominant genera in the NYS and EBS samples were *Pedospumella* and *Tetrahymena*, respectively (Figure S1). *Pedospumella* and *Paraphysomonas* were significantly more abundant in NYS estuaries than in the EBS area, while *Gymnodinium* was more abundant in the EBS area (Figure 2c, *p* < 0.05).

**Figure 3:**
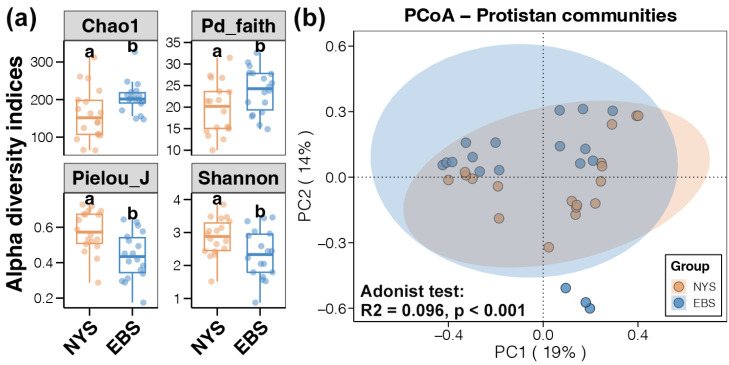
Differences in diversity of protist communities in the NYS and EBS. (**a**) Alpha diversity index (*t*-test, *p* < 0.05) of protist communities in the NYS and EBS, letters (a, b) above the small boxes in each boxplot indicate significant differences between groups. (**b**) PCoA plots comparing protist communities in the NYS and EBS based on Bray–Curtis distance (adonis test, *p* < 0.01).

Alpha diversity results showed that both Chao1 and Pd_faith indices of estuarine protists in the EBS area were significantly higher than those in the NYS area, in contrast, Shannon and Pielou_J indices of estuarine protists were significantly higher in the NYS area than those in the EBS area (Figure 3a, *t*-test, *p* < 0.05). This indicated that protist communities in the NYS estuaries had a more even species distribution, whereas those in the EBS estuaries contained more species with more complex phylogenetic relationships. PCoA analysis of Bray–Curtis distances of protist community compositions showed that the two axes explained 19% and 14% of the community differences, respectively. The distribution of data points indicated distinct clustering of protist communities in the NYS and EBS estuaries, demonstrating significant areal differences in their compositions (Figure 3b, adonis test, *p* < 0.01).

### 3.2. Unique and Shared Species in the Protist Communities

**Figure 4:**
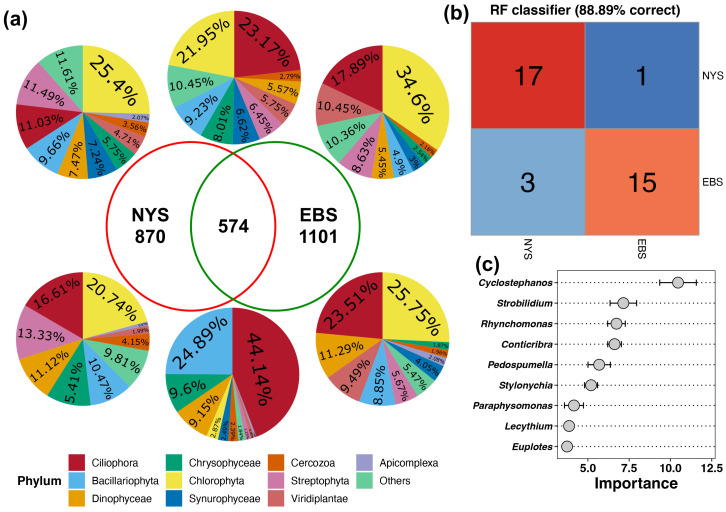
Differences in the composition of dominant protist species in the NYS and EBS. (**a**) Composition of unique and shared species in the NYS and EBS, with the top pie chart representing the composition ratio by species number and the bottom pie chart representing species abundance. (**b**) Predicted accuracy of random forest model based on protist families for classification the sample sources. (**c**) Contributions of different protist genera to the model’s differentiation of the NYS and EBS.

We used Venn analysis to identify the shared and unique species in the protist communities of the NYS and EBS estuaries. The results indicated that most species in the two areas were unique, with shared species constituting only about 10% of the total species. The Venn diagrams were plotted based on the number and abundance of the two types of species to analyze their composition ratios. The top pie chart represents the composition ratio by number, and the bottom pie chart represents the composition ratio by abundance (Figure 4a). In the NYS and EBS estuaries, 870 and 1101 unique protist ASVs were identified, respectively. The two areas shared 574 protist ASVs, which accounted for 22.6% of the total protist ASVs. These shared ASVs accounted for 95% of the entire protist community, with no significant difference in their proportions between the NYS and EBS areas (Figure S2).

The Venn diagrams showed that Ciliophora and Chlorophyta were the species most shared between the two areas, comprising 23.17% and 21.95% of the shared species. However, their abundance differed significantly, with Ciliophora accounting for 44.14% of the abundance and Chlorophyta accounting for 2.87%. In contrast, Chlorophyta had the most number and abundance of the unique species in both areas.

We used the protists data to build a random forest model to differentiate the regional origin of the water samples and to identify the protists that made key contributions to the different regional distinctions (Figure 4b,c). We used 18 samples each from the NYS and EBS areas for differentiation, of which 17 and 15, respectively, were correctly classified. The random forest model based on protist genera achieved an accuracy of 88.89% in distinguishing between NYS and EBS samples, suggesting significant geographic isolation between the two areas. The primary protists used for sample differentiation, ranked by their contribution to regional distinction, were *Cyclostephanos*, *Strobilidium*, *Rhynchomonas*, *Conticribra*, *Pedospumella*, *Stylonychia*, *Paraphysomonas*, *Lecythium*, and *Euplotes*.

### 3.3. Factors Affecting the Composition of Protist Communities

The DO in the EBS was significantly lower than that in the NYS, while NO_2_^−^ and NO_3_^−^ concentrations were significantly higher in the EBS. However, no significant differences in NH_4_^+^ and PO_4_^3−^ content, pH, salinity, and temperature were detected between the two areas (Figure S3).

Mantel tests indicated that the composition of protist communities was significantly associated with both environmental and geographic factors. However, Procrustes analysis indicated the association with environmental factors was higher than that with geographic factors (Table S1). db-RDA revealed that salinity, NH_4_^+^, and the geographic factors PCNM2 and PCNM3 were significantly associated with the composition of protist communities (Figure S4 and Table S2).

**Figure 5:**
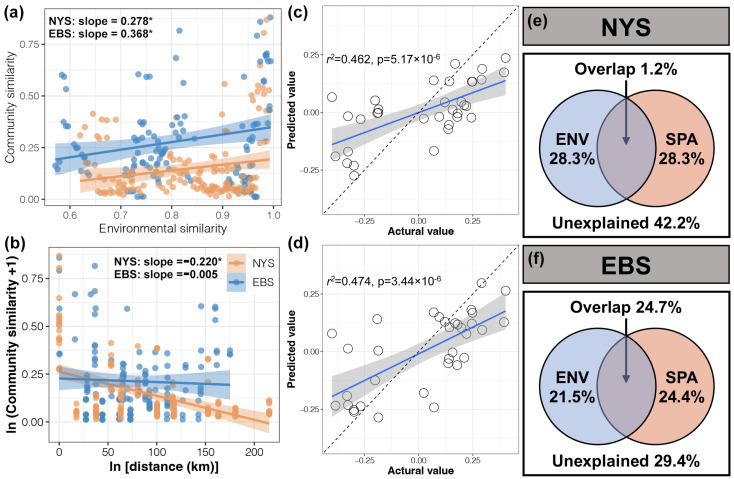
Linear regression of environmental similarity based on Euclidean distance (**a**) and geographical distance (**b**) with protist community similarity based on weighted Unifrac distance of the NYS and EBS estuaries, the “*” indicates *p* < 0.05. Predicted versus actual values of the effects of environmental factors (**c**) and geographic factors (**d**) on the composition of protist communities derived from the random forest model, circles indicate actual values, and the blue line indicates predicted values. VPA of protist communities in the NYS (**e**) and EBS (**f**) based on environmental and geographic factors.

We further compared environmental and geographic influences on the composition of protist communities in the NYS and EBS areas based on distance-dependent similarities (Figure 5a,b), and found that environmental influences on protist communities were stronger in the EBS area than in the NYS, whereas the opposite was true for geographic influences. A random forest model was constructed using environmental and geographical factors to predict changes in protist communities (Figure 5c,d). The results showed that the data points were distributed near the blue predicted regression line. Finally, VPA was used to analyze the contributions of environmental and geographic factors to the composition of protist communities in the NYS and EBS (Figure 5e,f). The results showed that the individual contributions of environmental and geographical factors to the composition of protist communities were relatively similar between the NYS and EBS. However, the overlap between environmental and geographic factors contributed significantly more in the EBS than that in the NYS.

### 3.4. Mechanisms Shaping Protist Community Assembly Processes

**Figure 6:**
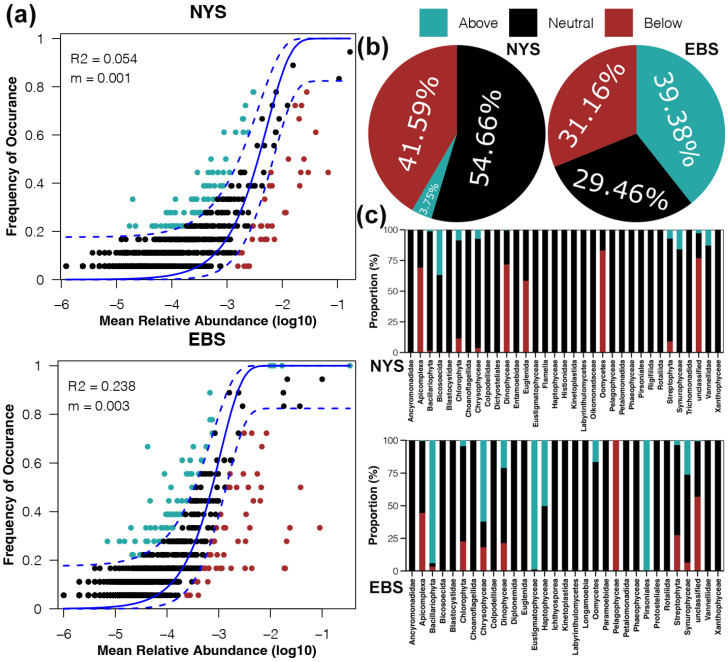
(**a**) Fit of the neutral community model (NCM) of protist community assembly in the NYS and EBS. The solid blue lines indicate the best fit to the NCM and the dashed blue lines represent 95% confidence intervals around the model predictions. R^2^ and m indicate the fit to the model and the species migration rate, respectively. (**b**) Proportions of the ASVs that occurred at higher or lower frequencies than predicted by the NCM. (**c**) Proportions of the taxa that occurred at higher or lower frequencies than predicted by the NCM at the phylum level.

We used the NCM to assess the proportion of species that conformed to the neutral site distribution. More species conforming to the neutral site distribution represents a greater contribution of stochastic processes to the shaping of protist communities. The neutral model indicated that the shaping of protist communities was primarily influenced by deterministic factors, and that stochastic processes contributed more in the EBS compared to the NYS (Figure 6a). In the NYS, species that did not conform to the neutral model were primarily of the Below type. In contrast, the proportions of Above and Below types were relatively similar and both were high in the EBS (Figure 6b). Further comparison at the phylum level revealed that in the NYS, Below type species belonged mainly to the Apicomplexa, Dinophyceae, Euglenida, and Oomycetes. In the EBS, Below type species belonged mainly to the Pelagophyceae, and Above type species were predominantly Bacillariophyta, Chrysophyceae, Eustigmatophyceae, and Pirsoniales (Figure 6c).

### 3.5. Differentiation of Protist Functional Diversity between Regions

**Figure 7:**
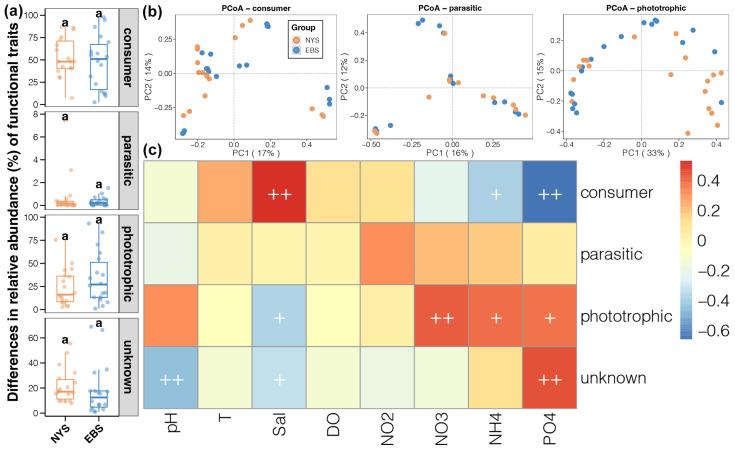
(**a**) Difference in the relative abundance of protist functional groups in the NYS and EBS. letters (a, a) above the small boxes in each boxplot indicate no significant differences between groups (**b**) PCoA analysis of geographic distribution of protist functional groups. (**c**) Spearman’s correlation analysis of protist functional groups with the environmental factors, “+” indicates 0.01 < *p* < 0.05, “++” indicates *p* < 0.01.

We assigned ASVs to three functional groups based on their taxonomic affiliation according to three main trophic modes: consumers, parasitic, and phototrophic. We found that consumers had the highest relative abundance, followed by phototrophic, and parasitic accounted for a very low relative abundance. No significant differences in the relative abundance of any functional group were observed between the NYS and EBS (Figure 7a). Further PCoA of the distribution of the three functional groups showed that consumers and phototrophs had significant geographic differences, while parasites did not (Figure 7b). Finally, results of Spearman correlation analysis of the relationships between protists and environmental factors showed opposing patterns for consumers and phototrophs: consumers were significantly positively correlated with salinity but negatively correlated with NH^4+^ and PO_4_^3–^ concentrations, while phototrophs exhibited the opposite pattern (Figure 7c).

## 4. Discussion

### 4.1. Comparison of Protist Community Composition between Regions

At the phylum level, Ciliophora was the predominant taxon in both the NYS and EBS. This finding is consistent with previous research on protists in temperate estuaries globally [31,32]. Ciliophora, which prefer small prey, play a crucial role in connecting small cells with higher trophic levels [33]. Bacillariophyta serve as a crucial food source for Ciliophora. Additionally, certain Bacillariophyta species form symbiotic relationships with specific Ciliophora. For example, Pseudovorticella coscinodisci can attach to the surface of Coscinodiscus wailesii, and the fluid flows generated by ciliary beating can substantially increase nutrient flux. Furthermore, the elevated prey availability surrounding host attachment sites benefits Pseudovorticella coscinodisci [34]. The high abundance of members of the Bacillariophyta may create ideal conditions for their survival. Ciliophore cells are large and possess comparatively intricate genomes, which results in a higher number of 18S rRNA gene copies per cell [35]. This could result in an overestimation of ciliophore abundance compared to protist phyla with smaller cell volumes. We found that the protist communities from the two areas exhibited greater dissimilarity at the genus level than at the phylum level. This disparity can be attributed to the more consistent composition of higher taxonomic levels, in which minor fluctuations within taxa do not significantly impact the overall community structure. Consequently, the microbial communities displayed greater similarity across different areas at higher taxonomic levels [36]. The most dominant genera in the NYS and EBS sample were Pedospumella and Tetrahymena, respectively. Pedospumella was defined by Boenigk’s group in 2010, and this name is of an entity that is currently accepted taxonomically [37,38]. A number of species of Tetrahymena have been described as being closely associated with a variety of metazoan hosts, including mussels [39]. Mussel farming in the Bohai Sea coastal area may provide favorable conditions for the growth and reproduction of Tetrahymena.

The Venn analysis revealed that although numerous species were unique to each location, the majority of the protist communities consisted of shared species that constituted a small fraction of the total species count. These shared species may have significant environmental adaptability and play essential ecological roles in both estuarine ecosystems, including nutrient cycling and maintaining biodiversity [40]. The protist communities in the NYS and EBS areas had the same structure, in that they were characterized by the presence of just a few species that played significant functional roles, whereas rare species served as a trove of biodiversity [41]. The high percentage of species number and abundance of chlorophytes unique to the NYS or the EBS area illustrate the distinct contrast between the chlorophyte communities in the two areas. As the primary producers in ecosystems, chlorophytes are significantly impacted by light [42]. The EBS estuaries are subjected to significant sediment deposition from the Yellow River, Liao River, and Hai River. Furthermore, the Bohai Sea’s semi-enclosed geographic characteristics lead to the retention of significant amounts of particulate matter, resulting in increased water turbidity [43]. Species that can maintain high growth rates in low-light environments, such as Scenedesmus obliquus, are more likely to inhabit and survive in such habitats [44].

### 4.2. Environmental Factors Had a Greater Influence on the Composition of Protist Communities in the Areas with Higher Nutrient Salt Concentrations

We found that the influence of environmental factors on the composition of protist communities was greater than that of geographic factors, potentially due to the significant environmental filtering effect of human activity. The disparities between environmental variables in the NYS and EBS can be attributed to anthropogenic stresses specific to each coastal region. The semi-enclosed EBS has been subjected to substantial contamination by densely populated and heavily industrialized coastal rivers, sewage systems, and offshore oil extraction [45]. The extended land reclamation operations have diminished the hydrodynamic conditions in the EBS area, rendering it more susceptible to the deposition of pollutants and increased levels of eutrophication [46]. Higher eutrophication levels in the aquatic environment can be identified by increased concentrations of nutritional salts, including NO_2_^−^ and NO_3_^−^ [47]. Eutrophication-induced enhancement of primary production and carbon accumulation generally results in decreased dissolved oxygen levels in coastal waters (Figure S2) [48]. These factors may explain the observed differences in the abundance of protists in the NYS and EBS. Previous laboratory experiments showed that the application of high concentrations of nutritional salts significantly increased the species abundance of protists while reducing evenness [49]. The environmental conditions in the EBS may enhance the dominance of species that are more suited to these conditions while suppressing the abundance of other species through competition for food and/or light. Conversely, studies of the NYS verified that the relatively low nutrient levels present in the region promoted greater species evenness [50]. However, the influences on the composition of protist communities in both areas had a component that could not be attributed to environmental or geographic factors, and biological factors likely were responsible [51]. Interactions between protists, such as competition, predation, symbiosis, or parasitism, can be classified as antagonistic or mutualistic, and these interactions likely influence species migration and nesting [52].

### 4.3. Deterministic Processes Governed the Assembly of the Protist Communities in the NYS and EBS Estuaries

Identifying the key ecological processes governing the assembly of protist communities in estuaries under anthropogenic pressure is critical for long-term watershed management [53]. Academicians now largely acknowledge that the assembly of the microbial community is influenced by both stochastic and deterministic processes, with their relative importance depending on the strength of selection and the rate of stochastic dispersal [54,55,56]. Previous studies have reported that the assembly of the bacterial community in estuarine environments relies primarily on stochastic processes [57,58,59,60], which is in contrast to our discoveries regarding protist communities. This discrepancy may be due to the generally larger size of protists compared to bacteria [61]. The size-plasticity hypothesis suggests that smaller organisms are more susceptible to diffusion processes than to environmental selection [62].

Our finding that environmental factors exerted a more significant influence on community composition than geographical factors provides evidence that the assembly of protist communities in both estuaries is predominantly driven by deterministic processes. Weaker hydrodynamic conditions in the Bohai Sea constrain microbial dispersal processes. Consequently, the assembly of local protist communities may have relied more on random births, deaths, and mutations of species—processes known as ecological drift, which belong to the stochastic processes within the framework of the neutral theory [63,64]. These activities further increased random fluctuations in the abundance of protists, resulting in a greater influence of stochastic processes on the assembly of EBS communities compared to NYS communities.

### 4.4. Consumers and Phototrophs Exhibited Biogeographic Patterns

Our investigation of the functional community structure of protists provided insights into the trophic profile of protists within the estuarine ecosystems. No significant differences in the abundances of consumers, parasites, or phototrophs were detected between the two locations, but consumers and phototrophs exhibited significant geographic differences. The observed strong positive connection between phototrophic organisms and NO_3_^−^ content suggests that the differences in phototrophic species at the two sites can be explained by the significant differences in NO_3_^−^ concentration. Because phototrophic organisms serve as food for consumers, they can indirectly impact the community structure of consumers, which could explain the observed differences between the protist communities in the NYS and EBS estuaries [65].

Consumers can prey on various eukaryotes, bacteria, and fungi [66]. The dominant abundance of consumers in the study area suggests that their predatory function plays a significant role in regulating the biomass, activity, and structure of bacterial and fungal communities [67]. The abundance of phototrophs was the second highest, only after consumers, potentially due to intense predation by consumers, which enhances nutrient cycling and may limit their biomass [30]. The absence of significant geographic variations of the parasitic species may be because many parasites have multiple hosts [68]. Moreover, certain life stages of parasitic organisms, such as spores and cysts, possess a remarkable capacity to adjust to environmental conditions [69], enabling them to disperse over vast distances via water currents or the movement of their hosts. For example, apicomplexans exhibit a great level of diversity in their life cycles, with morphologically distinct stages in one or more hosts, their versatile metabolic capabilities reflecting the capacity to survive and grow in different hosts and varying niches [70]. Finally, some phototrophic species are actually mixed trophic and alternatively behave as consumers [71]. Further research is needed to more comprehensively categorize and understand the relationships between protists in different functional groups.

## 5. Conclusions

In this study, we compared protist communities in two temperate estuary habitats that experience varying degrees of anthropogenic stresses. The composition and abundance of protist communities differed significantly between the two areas. Environmental and geographic factors collaboratively influenced the composition of protist communities. The EBS area experienced higher nutrient salt concentrations, and the composition of protist communities was more influenced by environmental factors compared to the NYS. Deterministic factors were the primary drivers shaping protist community assembly in both areas, and stochastic processes contributed more in the EBS area compared to the NYS area. Phototrophic and consumer communities, which were significantly correlated with environmental factors, exhibited distinct geographic distributions. In summary, protist communities exhibited distinct responses to varied degrees of anthropogenic stresses in the studied estuaries. Considering the limitations of our data, future studies should focus on long-term monitoring of protist communities in estuaries to clarify the impacts of human activities on their composition and assembly over time.

## Data Availability

The data presented in this study are available on request from the corresponding author. The data are part of an ongoing study.

## Supplementary Materials

The following supporting information can be downloaded at: https://www.mdpi.com/article/10.3390/microorganisms12081618/s1, Figure S1: The relative abundance of dominant genera in NYS and EBS; Figure S2: Boxplots illustrating the difference between relative abundance of shared species in NYS and EBS; Figure S3: Boxplots illustrating the difference between environmental variables of in NYS and EBS; Figure S4: RDA plots depict the influence of environmental factors and geographic factors on the composition of protist communities in NYS and EBS; Table S1: Mantel test and Procrustes analysis for protist communities with environmental and geographic factors; Table S2: Correlations between environmental and geographical factors with protist communities.
