# Supplementary material for: Comparing the Environmental Influences and Community Assembly of Protist Communities in Two Anthropogenic Coastal Areas
Source: Microorganisms. 2024 Aug 8;12(8):1618. doi: 10.3390/microorganisms12081618 (PMC11356250; doi:10.3390/microorganisms12081618)

Comparing the Environmental Influences and Community Assembly of Protist Communities in Two Anthropogenic Coastal Areas

Wenwen Qiao [1], Hongbo Li [2], Jinyong Zhang [2], Xiaohan Liu [2], Ruofei Jin [1,]* and Hongjun Li [2,]*

[1] Key Laboratory of Industrial Ecology and Environmental Engineering (Ministry of Education), School of Environmental Science and Technology, Dalian University of Technology, Dalian 116024, China; wenwen020416@163.com

[2] State Environmental Protection Key Laboratory of Coastal Ecosystem, National Marine Environmental Monitoring Center, Dalian 116023, China; hbli@nmemc.org.cn (H.L.); jyzhang@nmemc.org.cn (J.Z.); xhliu@nmemc.org.cn (X.L.)

* Correspondence: jruofei@dlut.edu.cn (R.J.); hjli@nmemc.org.cn (H.L.)

Table S1. Mantel test and Procrustes analysis for protist communities with environmental and geographic factors.

Factors	Mantel test		Procrustes analysis	
	R^2	*p*-value	M^2	*p*-value
Environmental	**0.210**	**0.004**	**0.791**	**0.001**
Geographical	**0.234**	**0.003**	0.946	0.270

Table S2. Correlations between environmental and geographical factors with protist communities.

Factors	R^2	*p*-value
pH	0.007	0.909
T	0.089	0.211
Sal	**0.397**	**0.001**
DO	0.116	0.139
NO2	0.142	0.079
NO3	0.058	0.359
NH4	**0.293**	**0.004**
PO4	0.149	0.056
PCNM1	0.032	0.588
PCNM2	**0.248**	**0.014**
PCNM3	**0.611**	**0.001**
PCNM4	0.039	0.551

Figure S1. The relative abundance of dominant genera in NYS and EBS.

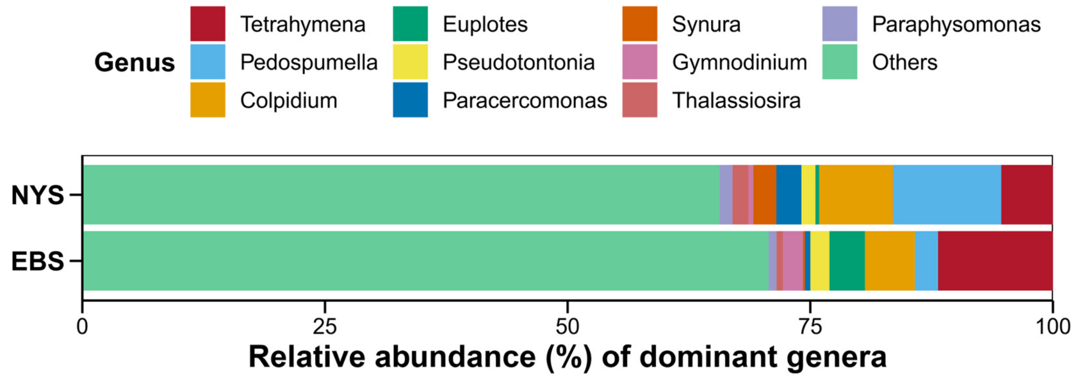

Figure S2. Boxplots illustrating the difference between relative abundance of shared species in NYS and EBS.

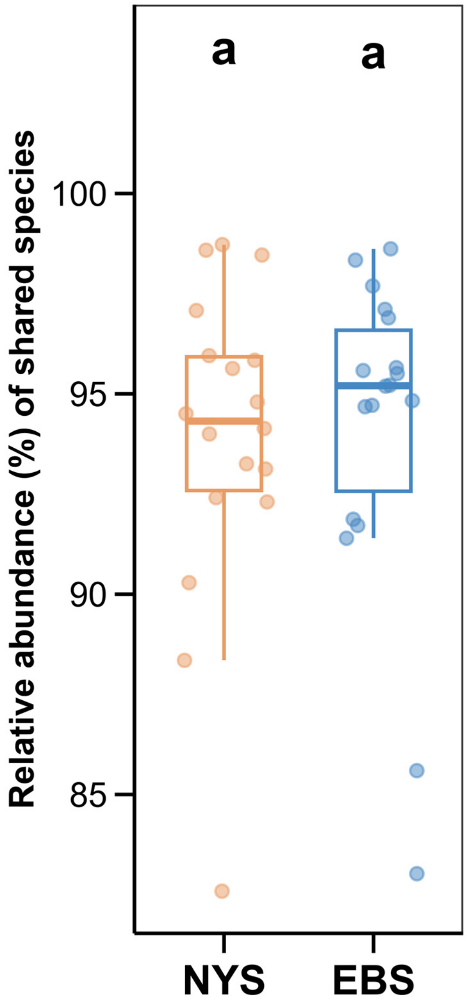

Figure S3. Boxplots illustrating the difference between environmental variables of in NYS and EBS, different letters (a, b) above the small boxes in each boxplot indicate significant differences between groups.

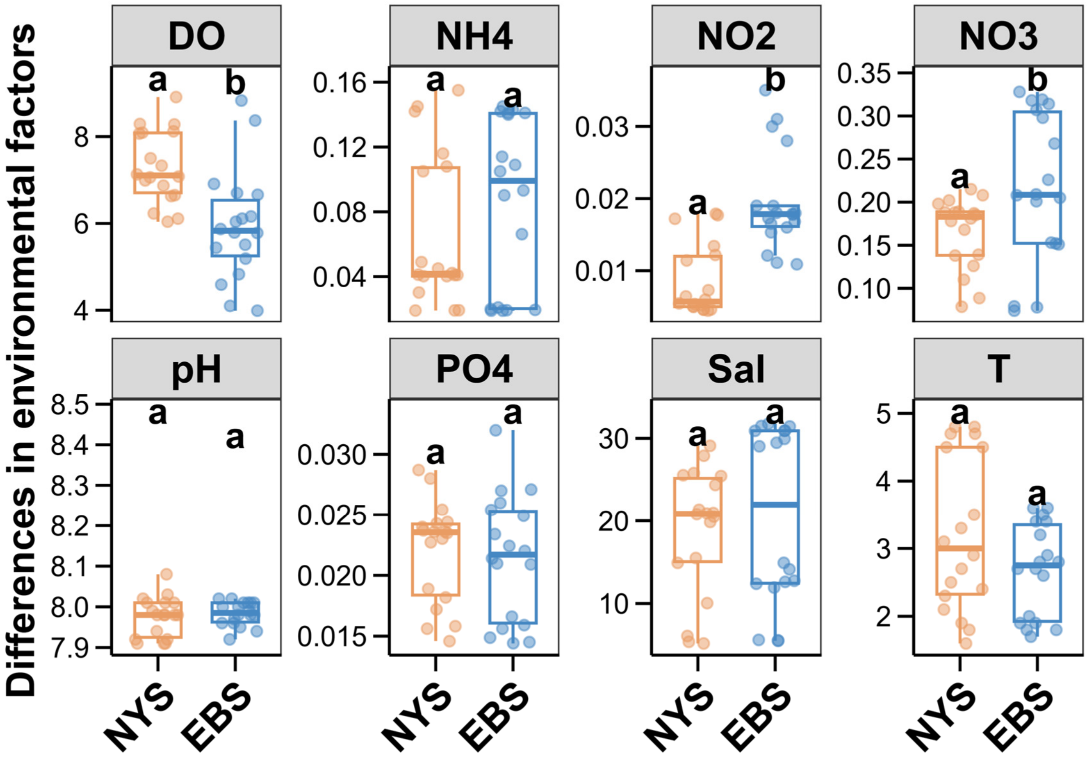

Figure S4. RDA plots depict the influence of environmental factors and geographic factors on the composition of protist communities in NYS and EBS.

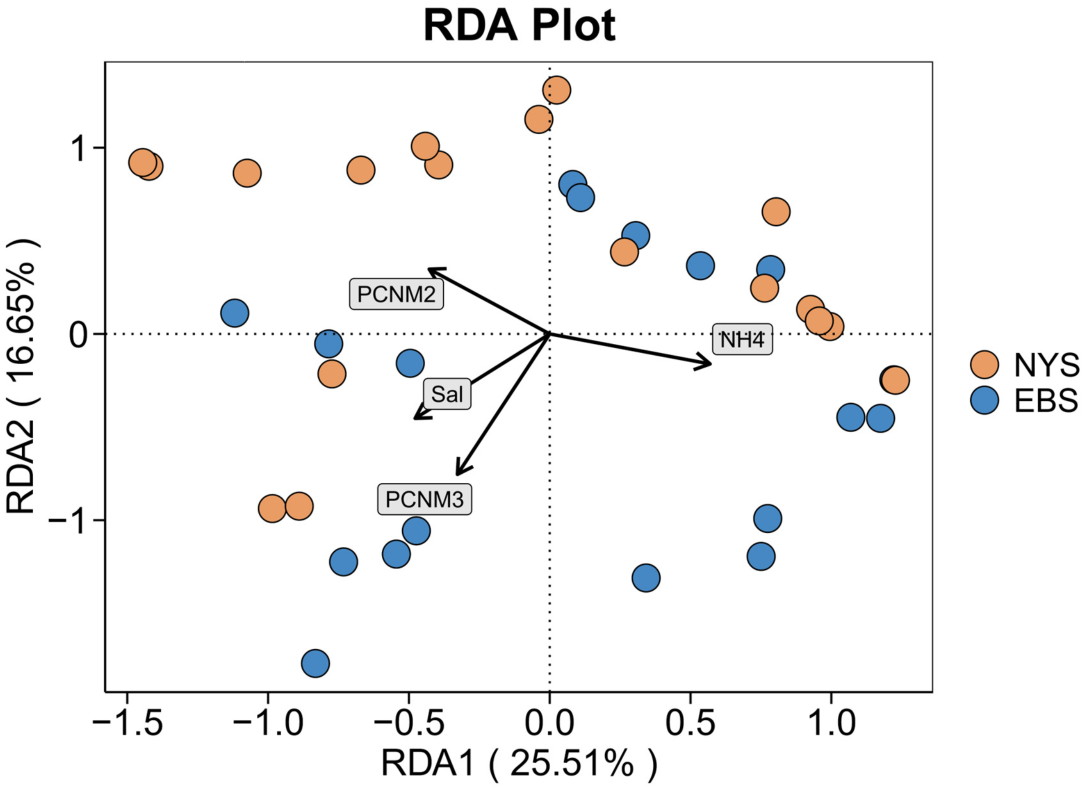

Supplement: Supplementary file 1 [file microorganisms-12-01618-s001.zip › microorganisms-3145241-supplementary.pdf]
